# Findings from the patch augmented rotator cuff surgery (PARCS) feasibility study

**DOI:** 10.1186/s40814-021-00899-9

**Published:** 2021-08-20

**Authors:** Jonathan A. Cook, Mathew Baldwin, Cushla Cooper, Navraj S. Nagra, Joanna C. Crocker, Molly Glaze, Gemma Greenall, Amar Rangan, Lucksy Kottam, Jonathan L. Rees, Dair Farrar-Hockley, Naomi Merritt, Sally Hopewell, David Beard, Michael Thomas, Melina Dritsaki, Andrew J. Carr

**Affiliations:** 1 Nuffield Department of Orthopaedics, Rheumatology and Musculoskeletal Sciences, University of Oxford, Oxford, UK; 2 Nuffield Department of Primary Care Health Sciences, University of Oxford, Oxford, UK; 3 National Institute for Health Research (NIHR) Oxford Biomedical Research Centre, Oxford, UK; 4 The James Cook University Hospital, South Tees Hospital NHS Foundation Trust, Middlesbrough, UK; 5 Frimley Health NHS Foundation Trust, Frimley, UK

**Keywords:** Rotator cuff tear, Feasibility study, Shoulder surgery, Tissue scaffold, Surgical mesh, Dermal matrix, Patch, Randomised trial

## Abstract

**Background:**

A rotator cuff tear is a common disabling shoulder problem. Symptoms include pain, weakness, lack of mobility and sleep disturbance. Many patients require surgery to repair the tear; however, there is a high failure rate. There is a pressing need to improve the outcome of rotator cuff surgery. The use of patch augmentation to provide support to the healing process and improve patient outcomes holds new promise. Different materials (e.g. human/animal skin or intestine tissue, and completely synthetic materials) and processes (e.g. woven or a mesh) have been used to produce patches. However, clinical evidence on their use is limited. The patch augmented rotator cuff surgery (PARCS) feasibility study aimed to determine the design of a definitive randomised controlled trial (RCT) assessing the effectiveness and cost-effectiveness of a patch to augment surgical repair of the rotator cuff that is both acceptable to stakeholders and feasible.

**Methods:**

A mixed methods feasibility study of conducing a subsequent RCT. The project involved six stages: a systematic review of clinical evidence; a survey of the British Elbow and Shoulder Society’s (BESS) surgical membership; a survey of surgeon trialists; focus groups and interviews with stakeholders; a two-round Delphi study administered via online questionnaires and a 2-day consensus meeting.

**Results:**

The BESS surgeons’ survey identified a variety of patches in use (105 (21%) responses received). Twenty-four surgeons (77%) completed the trialist survey relating to trial design. Four focus groups were conducted involving 24 stakeholders. Twenty-nine (67% of invited) individuals took part in the Delphi. Differing views were held on a number of aspects including the appropriate patient population for trial participation. Agreement on the key research questions and the outline of two potential RCTs were achieved through the Delphi study and the consensus meeting.

**Conclusions:**

Randomised comparisons of on-lay patch use for completed rotator cuff repairs, and bridging patch use for partial rotator cuff repairs were identified as areas for further research. The value of an observational study to assess safety concerns of patch use was also highlighted. The main limitation was that the findings were influenced by the participants, who might not necessarily reflect all stakeholders.

## Key messages regarding feasibility

- *What uncertainties regarding feasibility existed prior to the study?* Whilst randomised trials of rotator cuff repair surgery have been conducted, there remained uncertainty about how a randomised trial evaluating patch use should be designed. Specific areas needing clarification were the patient population, which patches should be evaluated, the intervention and control groups, the associated surgical technique and the acceptability of such a trial to stakeholders, particularly patients and surgeons
- *What are the key findings on feasibility from this study?* We identified a variety of patches for rotator cuff repair are available and in clinical use, although few have published evidence for their clinical effectiveness. The need for a RCT of the clinical effectiveness and cost-effectiveness of patch augmented rotator cuff repair was confirmed as was its acceptability to key stakeholders and its feasibility.
- *What are the implications of the findings on the design of the main study?* Two different randomised comparisons of interest were identified (1. on-lay patch use where rotator cuff repair has been completed and 2. bridging patch use for partial rotator cuff repairs) with an outline of the respective design proposed. The value of a registry for assessing safety of specific patches was identified.

## Background

### Clinical background

A rotator cuff tear is a common disabling shoulder problem. Symptoms include pain, weakness, lack of shoulder mobility and sleep disturbance. Initial management of rotator cuff tears is conservative and includes rest with simple pain management through paracetamol and non-steroidal anti-inflammatory drugs, along with physiotherapy and corticosteroid injections. Many patients will require surgery to repair their rotator cuff tear; however, there is a high failure rate between 25 and 50% within 12 months has been observed [1–4]. A number of surgical approaches have been developed to improve the outcome of surgery, but unfortunately, these have been largely unsuccessful [5–7]. The use of patch augmentation to provide support to the healing process and improve patient outcomes holds new promise. Patches have been made using different materials (e.g. human/animal skin or intestine tissue, and synthetic materials) and processes (e.g. weaving). Within this paper, we use the term ‘augmentation’ to refer more broadly to the use of a patch in rotator cuff repair irrespective of the specific usage. Augmentation can be carried out in two main ways—on-lay (placing the patch on top of a completed repair) and bridging (using it to fill a defect which the repair could not address).

Over 20 patches have received regulatory approval for use in surgical repair of the rotator cuff in the USA and/or by an EU-notified body at the time of study design [8–10]. A number of centres in the UK were using patches in rotator cuff repair for private and/or NHS patients at the time of study set-up. Patches currently in use reflect different materials and original purposes. Examples include the GraftJacket^TM^ (made from human cadaver dermis, originally developed for rotator cuff repair and available in different sizes and thicknesses), LARS ligament^TM^ (completely synthetic material originally developed for anterior cruciate ligament reconstructions and available in various versions including specifically for rotator cuff repair) and Permicol^TM^ (made from pig dermis and originally developed for hernia repair, latter a version for rotator cuff was produced called the Zimmer Collagen Repair Patch™). There appeared to be increased use of a patch to augment rotator cuff surgery.

The use of patches has not been without negative impact upon patients. One patch (Restore Orthobiologic Implant^TM^, a porcine based patch) was withdrawn from the market following clinical studies that identified a severe autoimmune response [10, 11]. In addition to any safety concerns, the use of a patch, if not effective, is a waste of precious resources in terms of staff, time and the cost of the implant.

There is a real need to improve surgical options for rotator cuff repairs and to improve outcomes for patients has been demonstrated [4, 6, 12]. The James Lind Alliance Priority Setting Partnership (PSP) for Surgery for Common Shoulder Problems brought together patients, carers and clinicians to identify the ongoing important treatment uncertainties related to shoulder surgery [5]. Four of the top ten uncertainties for common shoulder problems concerned care of patients with rotator cuff tears.

A feasibility study was necessary to address all these concerns. However, an unnecessarily long feasibility study could miss the optimal timing for evaluating this innovation in a surgical trial, as stated in Buxton’s law: ‘It’s always too early for a rigorous evaluation until suddenly it’s unfortunately too late’ [13]. For a surgical trial be successfully conducted, and to have impact upon clinical practice, buy-in from the key stakeholder groups is needed. A multistage mixed-methods research study was used to address the uncertainties (such as which patches should be evaluated, and for whom) related to the conduct of a randomised controlled trial (RCT) of patch-augmented rotator cuff surgery, and to seek consensus on the design of such as study.

### Aim and objectives

The aim of the PARCS study was to determine the design of a future definitive RCT, assessing the clinical effectiveness and cost-effectiveness of a patch to augment surgical repair of the rotator cuff that is both acceptable to stakeholders and feasible.

The study objectives were to (1) review existing evidence to identify candidate patches for use in a RCT and the evidence relating to their clinical use; (2) ascertain current NHS clinical practice relating to patch use; (3) explore the acceptability of the proposed trial stakeholders; (4) assess the feasibility of a trial of patch-augmented rotator cuff repair; (5) achieve consensus on the key RCT design elements of a definitive RCT; (6) confirm the scope of the health economic evaluation required and (7) identify areas for further research related to patch-augmented rotator cuff surgery.

## Methods

### Overview

**Fig. 1:**
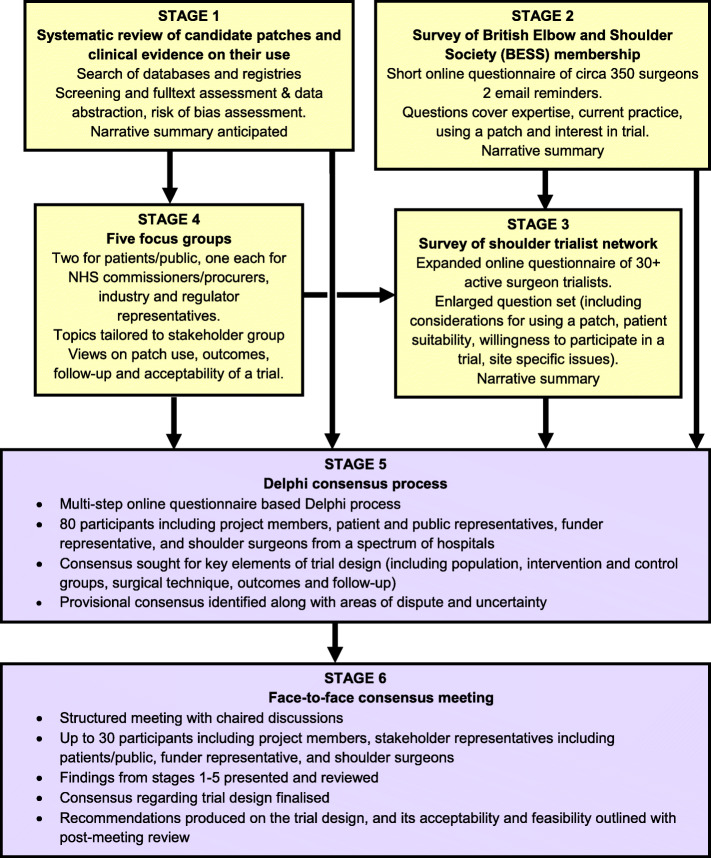
Patch augmented rotator cuff repair study (PARCS) feasibility study overview

PARCS was a mixed methods feasibility study (see Fig. 1 for an overview). It involved six stages: (1) a systematic review of clinical evidence; (2) a survey of the surgeons British Elbow and Shoulder Society (BESS); (3) a survey of surgeon trialists; (4) focus groups and interviews with stakeholders; (5) a two-round Delphi study administered via on-line questionnaires and (6) a two-day consensus meeting. The various stakeholders (including patients, surgeons and representatives from industry) were involved across the six stages. This manuscript summarises the methods and main findings of stages 2-6 along with the overall study findings. The protocols for the study (feasibility study and systematic review) are available elsewhere [14, 15]. More extensive details of the results of stages one and two are published elsewhere [16, 17] as is the full study report [18].

### Patient and public involvement

PPI representatives were involved in the study design and two were grant holders, and contributed to the project management. Two additional PPI representatives were involved in the management group. The study was set-up intentionally to seek PPI input in the stages of the research to inform the design and conduct of a future study. One impact of the PPI involvement was the implementation of the Delphi study was altered on the basis of feedback from one of the PPI representatives.

### Surveys

An invitation to complete an online survey was sent to the surgical membership of BESS in April 2017, and could be completed until August 2017. The survey questions covered respondent demographics, experience with patches, indications for patch augmentation and willingness to be involved in a RCT of patch augmented rotator cuff surgery. A second survey was directed at surgeons who had taken part in previous large multicentre UK shoulder trials. It focused upon trial specific implementation issues. The statistical analysis of the surveys was descriptive only.

### Qualitative study

Five focus groups were conducted which included participants from the three key stakeholder groups (patients and carers with experience of shoulder problems, NHS Research Ethics Committee members, NHS related administration and research support staff and industry representatives) were conducted. The aim was to access a broad range of views and opinions on the acceptability of the use of patches in the augmentation of rotator cuff repair within the context of a RCT. The two focus groups with patients and carers were held in different locations in England: Oxford and Middlesbrough. During each focus group session, the aim of the PARCS study, and focus groups specifically, were briefly introduced (facilitated by project team member NM). Participants were asked to consider a number of key issues, scenarios or vignettes. These included information about the possible trial design options, such as different patch types available and their acceptability; the choice of comparative study arms; most appropriate outcome measures and data collection methods. The focus group transcripts were analysed by two PARCS team members, whilst data collection was in progress, and the analysis was conducted using a thematic analysis approach. The following steps were followed:

1. Analysts familiarised themselves with the transcript.
2. One analyst (CC) initially coded the transcripts by hand, both deductively (guided by themes included in the focus group topic guides) and inductively (allowing unanticipated themes and sub-themes to emerge). The first three transcripts were also independently coded by a second analyst (JCC). The codes were subsequently compared, discussed and agreed with the first analyst. The emphasis of the analysis was on the acceptability of the proposed trial and on factors that might influence such acceptability.
3. Analysts reviewed the coding and agreed a working thematic framework. This was applied to subsequent transcripts using the QSR NVivo 10 software and evolved as analysis progressed. In accordance with the study design objectives, the themes were grouped into PICO elements, i.e.: their relationship to Patient population, Intervention, Control and/or Outcome (including timing of measurements). The framework also included practical considerations to take forward.

### Consensus process

A two-stage online Delphi study was conducted to develop a consensus on the best way to design a clinical trial of patch-augmented rotator cuff surgery. It was informed by the results of the systematic review, surveys and qualitative work (stages 1-4 of the feasibility study). Based on feedback received from the patient representatives on the project team, the initial round was sent to only the non-patient stakeholders (i.e. surgeons, physiotherapists, industry and researchers). Participants involved in stages two to four of the PARCS study were invited to take part in stage five, according to stakeholder group. Patients and public representatives were involved in the second round only (a third round was not anticipated to be necessary once the initial round had been outlined). An email was sent to each participant containing a personalised link that enabled access for convenient survey completion. For a subset of potential participants, paper copies were sent as per the participants’ preference, or a generic link was sent to a specific group of stakeholders. Under both rounds of the Delphi survey, non-responders received a maximum of two reminder messages. During completion of the first round, survey participants were asked their stakeholder group, and their place of work (professional stakeholders only). Data was summarised for data analysis purposes using Excel.

The last stage of the study was a 2-day consensus meeting with stakeholder representatives and project members held on the 29-30 January 2019. Findings from stages one to five of the PARCS study were received and consensus on the feasibility and acceptability of a RCT to address patch use for rotator cuff repair, and the basic design of such a study was sought. Ahead of the consensus meeting, participants were sent a proposal of a trial scenario for consideration, based on the results from the Delphi study. Patient and public representatives were reimbursed for expenses and compensated for their time [19]. The meeting was structured to ensure key areas of uncertainty and disagreement were reviewed and discussed. Consensus on key elements of the trial design were sought, namely, patient eligibility, intervention and control definitions, surgeon requirements, outcomes and target difference. Participants were selected for invite based on their perspectives and experience to ensure a variety of representation. For example, surgeons who do currently use patches to augment rotator cuff repair were invited along with those who do not use them but would potentially be willing to do so for a trial. Previous stages of the feasibility study informed draft guidance, options and recommendations for a RCT assessing patch-augmented rotator cuff surgery. A post-meeting report was drafted and circulated to participants for their review and comments.

## Results

### Surveys

For the BESS survey, 105 (21%) responses were received, with over half (61, 58%) stating that they had used a patch to augment rotator cuff surgery, 70% of which, had undertaken an augmented repair within the last 6 months. A wide surgical experience in augmentation was reported, ranging from one to 200 implanted procedures. However, most surgeons reported low volume usage, with a median of five rotator cuff augmentation procedures performed. At least ten different products were reported as having been used. Most of the patches were derived from decellularised dermis tissue, although porcine derived and synthetic based patches had also been used. Only 3-5% (3-5) of respondents stated they would undertake an augmented repair for small tears across ages, whereas 28-40% (29-42) and 19-59% (20-61) would do so for large or massive tears respectively. Patient age seemed more relevant for those with large and massive tears regarding assessing patient suitability. Half (52) of the surgeons reported an interest in taking part in a RCT evaluating the role of patch augmentation for rotator cuff surgery, with a further 22% (23) of respondent’s undecided.

For the surgeon trialists survey, 24 (77%) responses were received from those invited to take part. Twenty (83%) used a patch or would be willing to do so in a trial. The importance of assessing the integrity of the subscapularis prior to the potential use of a patch were evenly split with 11 (55%) responding that they would consider the state (i.e. whether it was intact, partially torn or fully torn). Typical patch was evenly split between the two main approaches, ‘on-lay’ (5, 45%) and ‘bridging’ (6, 55%). Responses for age and tear size, and including revision operation for the 2 and 3 arm trial scenarios were very similar. With regards to the running of a definitive trial, almost all trialists (17, 85%) supported having a standardised operative technique and 19 trialists (95%) a standardised post-operative rehabilitation regime. Eleven trialists (55%) supported randomising in the operating room, and with 12 and 24 months follow-up considered agreeable by almost all trialists, 18 (90%).

### Qualitative study

The five focus groups involved 24 stakeholders (15 patients and carers, 4 industry representatives, 2 NHS Research Ethics Committee members and 3 Clinical Research Network representatives). All of the stakeholders who participated in the focus groups, supported patch use in rotator cuff repair surgery. They acknowledged the risk involved in receiving an implant like the patch, and advocated product safety monitoring during the trial. However, there was some discrepancy amongst the stakeholders about what the patient population, intervention and control arms should be in the trial. Some indicated they would be unwilling to participate in a trial with a ‘no patch’ control arm, if there was access to a patch available within routine care. The important outcome identified in the focus groups was improvement in pain and function and preventing further treatment.

### Consensus process

Of the individuals invited to the Delphi study, 29 (67%) participated. Twenty-three and 24 (including 3 PPI respondents) responses to rounds 1 and 2 were received. Initial agreement on five of six domains was met at round 2 amongst non-PPI participants, with patient eligibility begin the exception, 11 (52%). There was strong agreement for randomisation in theatre (18 (86%)), using the available patch at each site (17 (81%)), 2-arm design of repair with patch versus no patch (18 (86%)), collecting patient reported outcomes measures and imaging assessments (19, (90%)). The PPI respondents were supportive of proposals regarding patches, study comparison, randomisation in theatre and follow-up for 24 months by questionnaires. One had uncertainty about blinding the participant to patch use, and two were unsure about follow-up requiring return to the hospital. The initial proposal based upon the Delphi study was revised in light of the discussed at the consensus meeting at which 21 participants attended. The outline of two potential RCTs was developed. The first assesses the use of a patch as an on-lay for patients with a completed rotator cuff repair, and the second relates to patients with a partial rotator cuff repair using a bridging approach. The two comparisons could potentially be within one more comprehensive trial or conducted separately. Additionally, the need for an observational safety study was identified.

### Study proposals

**Fig. 2:**
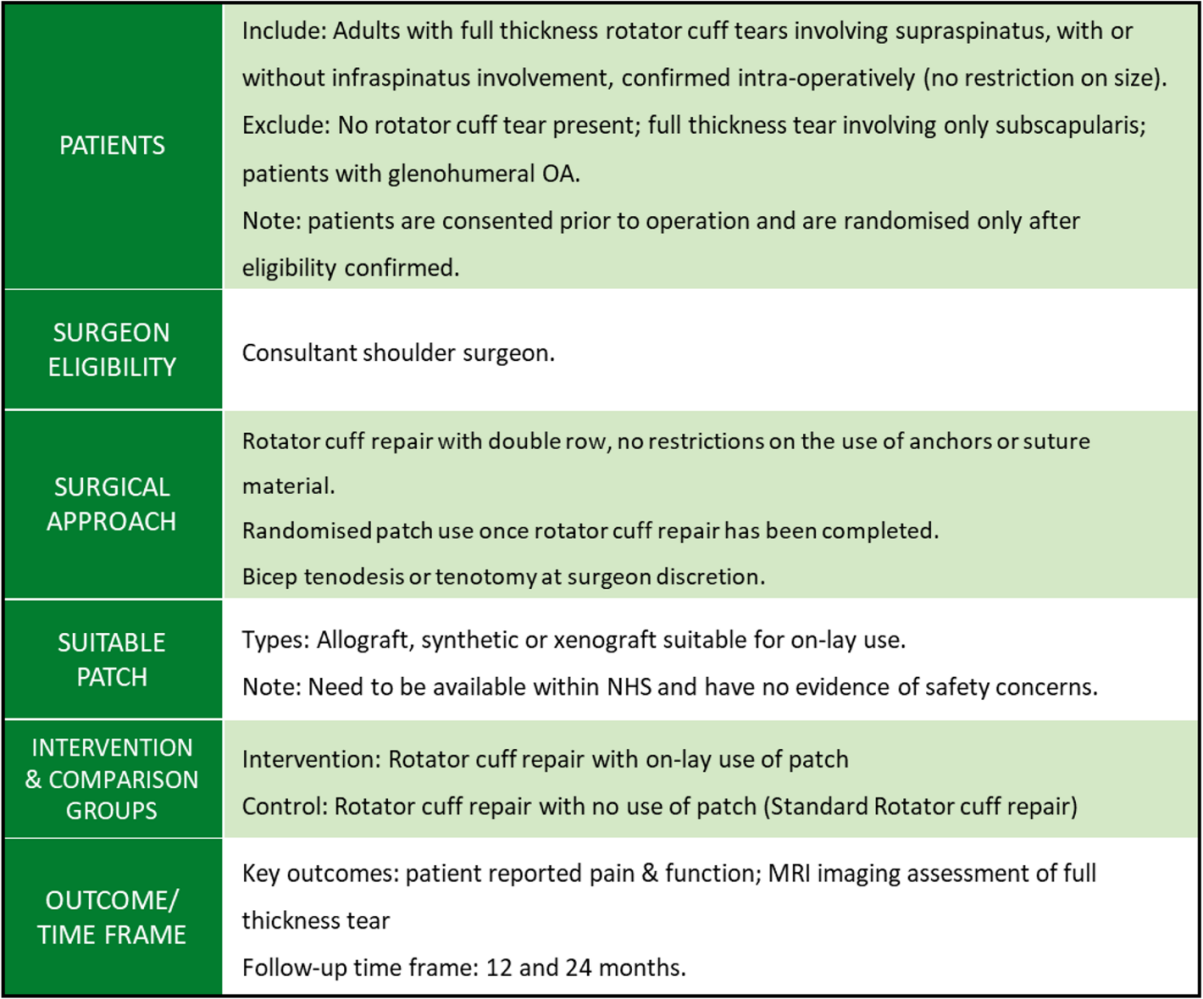
Randomised trial proposal using on-lay patch technique

**Fig. 3:**
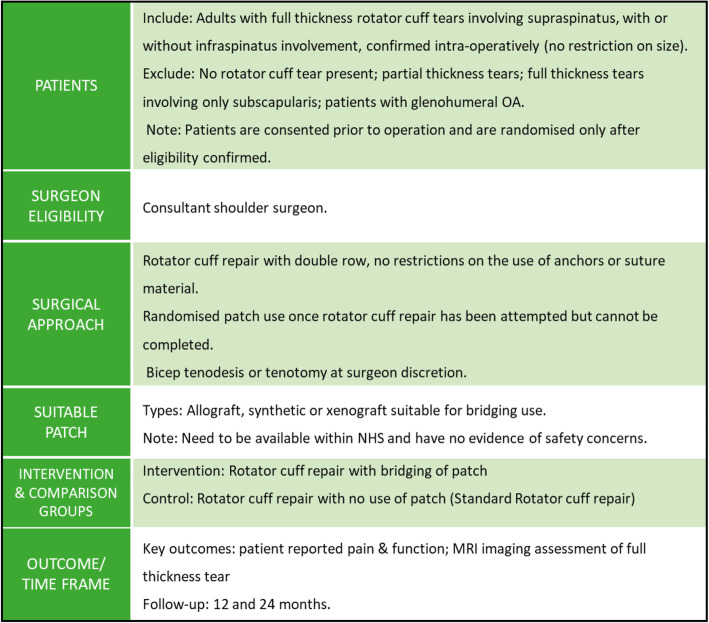
Randomised trial proposal using bridging patch technique

**Fig. 4:**
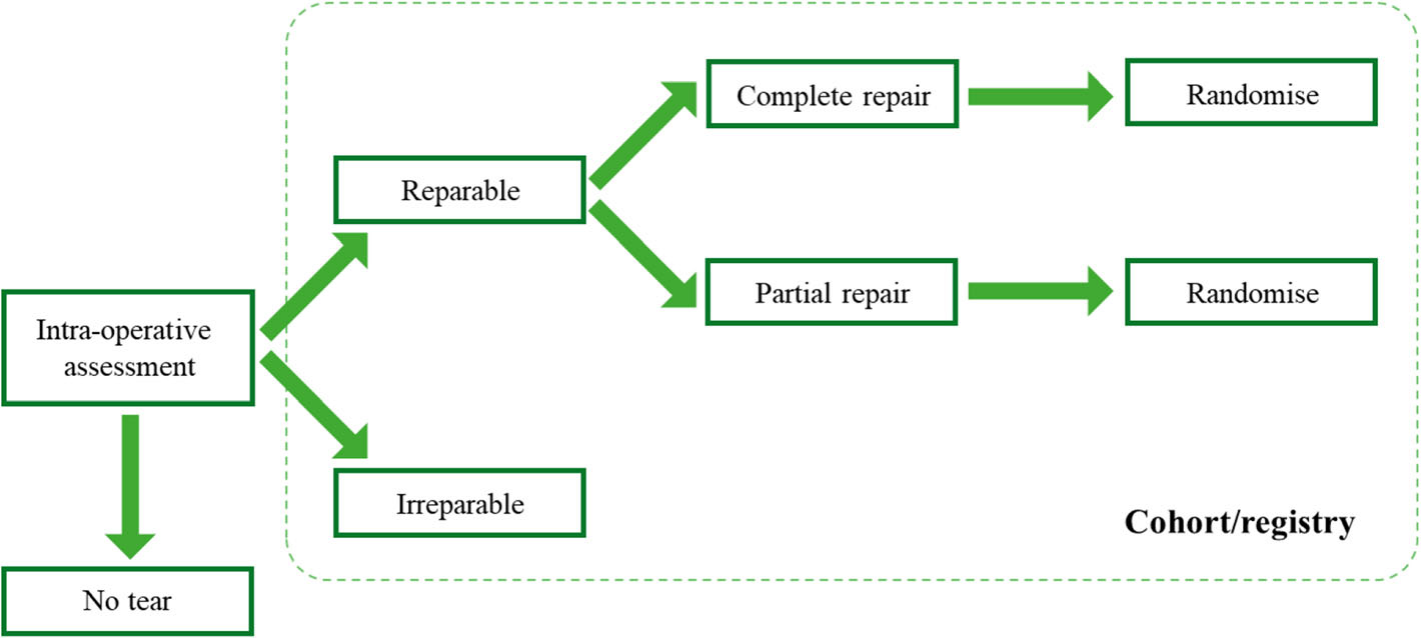
Proposed study patient eligibility pathway

An outline of the agreed study proposal is given in Figs. 2 and 3*.* Two areas for a RCT assessing the use of patches in rotator cuff surgery were identified. The first RCT seeks to assess the use of a patch as an on-lay for patients with a completed rotator cuff repair, and the second relates to use of bridging patches for patients with a partial rotator cuff repair. Figure 4 illustrates the patient flow, along with patients who potentially could contribute data to a registry to inform upon the safety of specific patches. The two randomised comparisons could potentially be within one more comprehensive trial or conducted separately.

## Discussion

### Summary of findings

This feasibility study has confirmed the need for a RCT of the effectiveness and cost-effectiveness of patch augmented rotator cuff repair. It has demonstrated that a trial would be both acceptable to key stakeholders and feasible. Across the five stages of the feasibility study involving stakeholder engagement, general support for further research on the use of patches was demonstrated throughout the groups, including the willingness to participate in a RCT. There were key challenges related to the implementation of such a trial, and decisions related to its conduct were identified and tackled. Given the variations in the patches and their current use, it is unlikely that a single study would be able to address all of the key research questions.

### Strengths and limitations of the work

The main strength of the PARCS feasibility study was the use of multiple methods to engage with all potential stakeholders to address the aim and objectives of the study. Objectives were addressed as intended. However, there were a number of limitations to the work. Inevitably the success of engagement with the stakeholder groups varied, with the most limited input from industry representatives. It might be argued that having representatives from all stakeholders groups involved is a strength even if the level of input might have been less than desired for some stakeholder groups. The response rate of the BESS membership survey was low, even if consistent with other surveys sent to this society or other similar surveys of such societies. Fewer participants took part in the Delphi study than originally hoped. It is therefore difficult to know how representative the findings are of each stakeholder groups’ views. However, there is confidence that those who participated, particularly the surgeons, would want to be involved in a trial.

Three studies which would progress the field were identified, including two RCTs. Ultimately, the value of the PARCS study will only be confirmed (1) if a RCT assessing the use of a patch in the UK NHS setting is attempted, and (2) if successful, how influential the study is for clinical practice.

### Key issues related to conducting a RCT

Patient eligibility provided the most disagreement in the study. A number of aspects were agreed on, such as the exclusion of patients with other shoulder problems and with clinically significant osteoarthritis. There was a variety of specific exclusion criteria proposed by individual respondents, with a range of views on age, muscle atrophy, tear size and having a previous rotator cuff repair. The relationship between patient eligibility and patch technique was noted by a number of participants. The final proposal reflects an inclusive approach whenever possible. The broad criteria were accepted, though the details were not fully resolved.

A variety of patches are available for clinical use, reflecting different materials, processes and designs [17]. The systematic review (see Baldwin et al.) [17] identified 28 different patches, defined as an implantable human, synthetic or animal material, used with the aim of improving tissue healing and/or patient outcome via some form of mechanical support. Of these, 22 could be classed as a product, and six were a tissue graft from either the patient, or (in one case) a cadaver. There was comparative evidence for only 12 different patches. The survey of the surgical BESS membership identified 13 different patches currently in use in the UK. Most are produced from decellularised human dermis, with the rest being made from porcine or synthetic materials. It is concerning that these two groups did not overlap fully; there are at least 2 patches (dCell and Leeds-Kuff) in current clinical use for which no clinical evidence was identified in the systematic review. No single type of patch could be considered to be either dominant in terms of use, or evidence in its favor. Some evidence suggested allograft and synthetic patches to improve the re-tear rate, and for synthetic patches to reduce pain. Mostly the evidence is non-existent, or too weak to draw even tentative conclusions.

With regards to running a large definitive trial of patch use for rotator cuff repair, it was clear that the use of a specific patch would be unwarranted, but a decision about which types of patch to allow within the study is of key importance. Any secondary evaluation of evidence, such as safety of the included patches would be advantageous. The need for a non-animal based patch in the trial design, particularly an alternative to a porcine-based patch, was noted.

The surgical use of patches in rotator cuff surgery falls broadly into two groups. During the on-lay technique, the patch is sutured on top of the tendon-to-bone repair, whereas in a bridging technique, the patch is sutured into the exposed area following a partial repair. Whilst to some degree, a partial repair can be anticipated in advance, this is not always the case, as the quality of the tissue is not entirely clear until the repair has been attempted. This became increasingly apparent as surgeon stakeholders were engaged through stages two to five. To address the two techniques, two RCTs were proposed; one for on-lay use and one for bridging. Different patch types and were thought by some stakeholders to be better suited to each approach.

Beyond this, there was overall support for flexibility for surgeons to conduct patch augmented rotator cuff repair according to their personal practice. A standardised post-operative regime was supported by the surgeon trialists in stage four of this feasibility study.

### Conducting a randomised controlled trial

Specific information related to how a RCT might be conducted was gained from the surgeon trialist survey, the Delphi study and the consensus meeting. The Delphi study and the consensus meeting showed most support for a two-arm trial of rotator cuff repair with and without patch use. Whilst differences between patch types were noted by a number of participants as being of interest, the difference in how patches are used appeared to be a higher priority for evaluation. This is reflected in the research recommendations of the study.

Randomisation during the operation was most supported, once the rotator cuff repair had been attempted. This has the benefit of confirming the presence and nature of a tear, and knowing whether the repair could be completed or not.

Participants in the surgeon trialist survey, Delphi study, and consensus meeting supported use of both a patient reported outcome measures and imaging as trial outcomes. There was support for 24-months follow-up. The timing of assessments within this period indicates support for an ‘early’ assessment around 4 months, followed by further assessments at 12 and 24 months. It was not viewed as necessary to have the same outcomes at every time point. Based upon the UKUFF trial results, most function gain (as measured with the Oxford Shoulder Score) appeared to have occurred by 12 months though there was further improvement between 12 and 24 months. Re-tearing or non-healing of the tear was observed in around 40% of patients at 1 year indicating this time-point was a pertinent one. The first 12-15 months post-surgery appears to be when most early re-tears occur though rates vary by age and tear size [1–4, 20, 21].

Many feasibility studies focus on issues such as demonstrating feasibility of randomisation, achieving sufficient recruitment and retention, confirming outcome measurement and sample size considerations [22]. The approach adopted here was quite different reflecting knowledge at the time of designing the study, previous and ongoing trials in this area conducted in the UK in the same setting which in our view substantially addressed these common concerns [4, 23]. Indeed, since conducting our feasibility study, a further UK study of rotator cuff repair for partial tears is due to commence this year [24]. Instead our focus was predominantly around the intervention, and patches, which could be used and when, evidence on their use, and the views of stakeholders regarding a trial of their use. These were the key uncertainties related to a similar scale UK study of patch use in rotator cuff surgery.

### Economic evaluation considerations

A future definitive RCT of patch use should consider embedding an economic evaluation of the patches under investigation in order to assess their cost to the National Health Service (NHS) UK as well as their benefits to patient health-related quality of life (HRQoL).

Little evidence was available to shape an RCT economic-based evaluation. Considerations about the types of data (e.g. inpatient and outpatient visits), frequency and intensity of patient data collection as well as the means of data collection have to be made when designing a prospective RCT. Resource utilisation not related to the surgery, such as use of non-NHS care to deal with daily activities as well as loss of income due to surgery are also recommended to be included in the patient data collection process. Finally, SF-36 although was the only HRQoL to be captured in the current literature, a future economic evaluation, in our view, needs to also administer the recommended by NICE EuroQol EQ-5D measure.

## Conclusions

Whilst several experimental and observational studies have demonstrated a decreased failure rate and improved outcome scores following augmented rotator cuff repair, rigorous clinical evaluation of this technology with long-term follow-up is currently lacking which prevents firm recommendations for practice. We identified that a variety of patches for rotator cuff repair are available and in clinical use, although the few have published evidence for their clinical effectiveness.

Areas for further research identified were randomised comparisons of on-lay patch use where rotator cuff repair has been completed, and of bridging patch use for partial rotator cuff repairs. The value of a registry was also highlighted.

## Data Availability

The data that support the findings of this study are available from the corresponding author pending reasonable request.

## Abbreviations

BESS: British elbow and shoulder society
EU: Europe/European
HRA: Health RESEARCH AUTHority
NHS: National health service
PICO: Patient intervention control outcome
RCT: Randomised controlled trial
SSC: Study steering committee
USA: United States of America
